# Planetary microbiology: microbes, planets, and the search for life

**DOI:** 10.1128/aem.00241-25

**Published:** 2025-09-30

**Authors:** Betül Kaçar

**Affiliations:** 1 Department of Bacteriology, University of Wisconsin-Madison 205263, Madison, Wisconsin, USA

**Keywords:** planetary microbiology, early microbial life, origins of microbiology, astrobiology

## Abstract

Life on Earth has been shaped by transformative microbial innovations and singularities that redefined planetary systems, from oxygenic photosynthesis to biological nitrogen fixation. These unique events, occurring only once in life’s tractable history, laid the foundation for the complex ecosystems and global biogeochemical cycles around us today. The Planetary Microbiology collection in *Applied and Environmental Microbiology* explores how modern microbiological tools reveal the origins, early evolution, and planetary impacts of these microbial breakthroughs. Spanning deep-time studies, extreme environments, and astrobiology, contributions in this issue link biosignatures and ancient microbes to the search for extraterrestrial life. By bridging molecular, ecological, and planetary scales, planetary microbiology illuminates life’s past while critically informing strategies for the environmental and exploration challenges of our future.

## EDITORIAL

Life on Earth has existed for nearly 4 billion years, and for most of that time, it was microbial (1). The diverse world we see around us today owes its entire existence to a few foundational events carried out by ancient microbes (2).

Life is a form of chemistry with memory. It solves problems and carries a memory of molecular solutions across generations. Over time, solutions that first arose in one part of biology can profoundly shape a seemingly unrelated domain through the network of ecological relationships, the meandering paths of variability and horizontal gene transfer, the endurance afforded by inheritance, and the complex coupling between organisms and the environments that they create and maintain.

Single molecular breakthroughs, like oxygenic photosynthesis, rippled forward across billions of years, entirely reshaping the planet’s biosphere (3). One of the earliest of these breakthroughs was the ability to convert carbon into an interactive energy reservoir suited to Earth’s environment (4). Since then, life has evolved a cascade of molecular strategies through various improbable encounters, capturing sunlight, extracting nitrogen from the atmosphere, using water as a source of electrons, and even hosting one microbe inside another, a relationship that gave rise to the complex cells we’re made of today (5). It is a fact: none of us would be here today if microbes hadn’t retained all of these breakthroughs across eons.

Events like these are singular in life’s history. Current evidence suggests that there is one origin of life (6), one origin of oxygenic photosynthesis (7, 8), one eukaryotic ancestor (9), and one biological way to fix nitrogen into ammonia (10). They are so rare and special that we refer to these events as “singularities” in the words of de Duve (11). Biological singularities appear to have happened once and only once, interspersed by vast distances of time and space, and to have no obvious equal, parallel or contemporary. There are no other survivors from these events to tell a tale of competition, victory or loss. We know of these singularities because they only have a single surviving example, each of which came to completely reshape our planet for every day that followed. Even in the succeeding billions of years, complex life co-opted and built upon these “super survivors”. Yet, despite this complexity, it did not (and perhaps could not) replicate or exceed them.

We have some understanding of the chronology of life on Earth (12), and yet, we still grapple with understanding how these foundational events occurred in the first place (13). How did these intricate interactions first emerge and expand on the planet? How did microbes coevolve with a changing planet? How did their metabolic evolution build the foundations of life as we know it? To truly grasp the origins of microbiology, we must rethink and reconceptualize it from the ground up. We must begin, as Lewis Carroll wrote, “at the beginning”. The true challenge is the reconstruction of the evolutionary milestones themselves and the study of their evolution and persistence (13). We must thus revisit life’s own record, a record written by microbes, however fragmented (14), if we are to confront one of the grandest challenges in microbiology.

In this special collection published in *Applied and Environmental Microbiology* (AEM), under the theme of Planetary Microbiology, we seek to do just that. The Planetary Microbiology collection challenges us to scale our exploration, to see life from the molecular to the planetary. Modern microbiological tools are transforming how we approach some of biology’s biggest unanswered questions. They help us to make sense of the origins and evolution of microbiology within the broader context of planetary processes. The ambition of the papers in this Planetary Microbiology collection is rooted in a deceptively simple question: how can modern microbiology help us traverse billions of years of macroevolutionary time?

Studies published in AEM’s Planetary Microbiology collection range from exploring non-thermodynamic factors such as hydrogen availability, growth temperature constraints, metabolic trade-offs, and hydrological dynamics that shape competition among thermophilic, hydrogen-oxidizing autotrophs (15), to state-of-the-art methods for culturing microbes at high pressure and temperature regimes (16), investigating Martian analog environments such as the Soudan underground mine (17), studying halophilic microbial adaptation to perchlorate-rich environments (18), and developing life-detection instrumentation and technologies using *Shewanella* strains from Blood Falls, Antarctica (19).

Together, these efforts emphasize that advancing both basic and applied microbiology is essential not only for elucidating microbial survival in analog worlds but also informing the development of detection systems that may one day interpret traces of life (biosignatures) on Mars (20), Europa (21), and beyond (22). Further, identifying and studying ancient Earth analogs remain indispensable for guiding astrobiological exploration and reconstructing the conditions that may support life elsewhere (23). In this context, Gonzalez-Henao and Schrenk bring forward an expanded perspective: that microbial biofilms and their extracellular polymeric substances ought to be regarded as more than mere survival strategies. Instead, extracellular polymeric substances are to be understood as structurally persistent systems that mediate mineral interactions and regulations, leaving behind distinctive, potentially long-lasting biosignatures susceptible to detection by planetary exploration instruments (24).

A paleontological study shows how microbial metabolism can drive planetary-scale change by revisiting the Lomagundi–Jatuli carbon isotopic excursion (25), a 2-billion-year-old anomaly captured by Earth’s carbonate rocks marked by an unprecedented enrichment in the heavier carbon isotope (^13^C) (26). Sumner frames the oxygenation of Earth’s atmosphere as “the most extensive chemical transformation of a planet’s surface by microbial life,” and in doing so, offers a crucial template for microbial astrobiology (25). This paper suggests that when we look for signs of life on Mars or even an exoplanet, we should not limit ourselves to seeking biomolecules. We must also seek evidence of the planetary transformations life might have impacted or altogether caused. As stated in a commentary by Prave in the collection, the study reframes the Lomagundi–Jatuli event through the lens of enzyme evolution and carbon substrates, providing a fresh take on the paleobiological record of Earth’s oxygenation (27).

To understand how Earth’s climate has evolved, it’s essential to study microbial life. Papers in the Planetary Microbiology collection reveal the deep connections between microbes and global environmental shifts, approaching early life by scaling down from the geological to the molecular level, leveraging the geochemical and genomic record. One study by Wannicke et al. revisits the evolution of nitrogen fixation in the rock record (10) by examining nitrogenase-driven isotope signatures under elevated carbon dioxide conditions (28). A study by Harrison et al. reevaluates RuBisCO’s potential to power life in carbon-stressed, dark, cold, and saline environments, conditions likely reflected elsewhere, such as on Enceladus and Europa (29). A third contribution retraces the evolutionary history of photosystems through refined molecular clock analyses, shedding light on how the machinery of light capture emerged in Earth’s early biosphere (30) . Finally, Harris et al. report the potential utility of archaeal lipid δ^2^H values as a hydrological proxy for extreme environments as well as extraterrestrial settings (31). Together, these studies highlight how tracing microbial signatures deepens our understanding of Earth’s climate history and informs the search for life beyond it.

### Bridging microbial histories with planetary futures

Microbes are dynamic; their metabolisms, ecologies, and biochemical toolkits have continually transformed with Earth’s changing environments, reshaping the planet and being reshaped by it (13). Microbiota have attenuated themselves to massive upheavals before, such as increases or decreases in available substrates and nutrients or dramatic shifts between marine and terrestrial niches. Organisms have had billions of years and countless generations of tinkering and have sampled sequence and environmental combinations that dwarf those typically employed in laboratory experiments.

Planetary microbiology is the study of microbial evolution across vast scales of space and time. In practice, it also represents the development of shortcuts to likely functional molecular and metabolic variants that are no longer widely expressed in modern organisms. What has changed today is our ability to track key aspects of these attenuations, which are indelibly imprinted in the histories of biosynthetic systems that have persisted through these challenges.

### Final remarks

Advances in our search for life beyond Earth are necessitating microbiologists to ask integrative questions and to reconceptualize microbial life’s past, present, and future within a planetary context. A recent special collection by the American Academy of Microbiology underscores this direction (32), urging microbiologists to consider how the origins and evolution of early microbial life bear directly on future climate challenges and extraterrestrial exploration. Central to this effort are questions such as: what are the planet-scale transformations mediated by microbes, and how did they emerge? What evolutionary innovations shaped life as we know it? How can ancient microbial interactions inform our strategies for detecting life elsewhere in the cosmos? Tackling these questions requires bridging scales from molecular machinery to planetary geochemistry, and leveraging diverse tools across biology (including molecular, computational, and synthetic), chemistry, and Earth sciences to trace life’s signatures across time and space.

As we prepare to send new missions to Mars, drill into Europa’s ice shell, fly through Enceladus’s plumes, and even read the atmospheric spectra of distant exoplanets, the lessons from this collection are timely. The danger isn’t just failing to find life. The danger is failing to recognize it when it’s there, possibly encoded in unfamiliar molecules or in strange patterns of minerals.

Understanding the origins and evolution of microbial life is a necessity for understanding life in the universe. For if we hope to find life elsewhere, we must first reveal the most beautiful story ever told: the story of microbial evolution on Earth. This is not to say that efforts along these lines will remain focused on understanding the past. The greatest value may lie in exploring sequence and functional possibilities for dealing with uncertainties of our own future (33). We thus start from here. We have a long way to go. As Galadriel wisely remarks in *The Rings of Power*, “Even the smallest person can change the course of the future.” The Planetary Microbiology collection adapts this sentiment: Even the smallest molecule (perhaps especially the tiniest of them all) holds the power to change the course of a future that is yet to be written.
